# Factors Associated With Transition of Care in Patients With Chronic Non‐Communicable Diseases: Cross‐Sectional Study

**DOI:** 10.1002/nop2.70494

**Published:** 2026-03-31

**Authors:** Mardhjorie dos Santos Seidler, Letícia Flores Trindade, Larissa Berghetti, Letícia Y. Castro, Francini de Oliveira Rodrigues, Cristina Lavareda Baixinho, Manuel Portela Romero, Amanda Caroline Mélo da Rosa, Adriane Cristina Bernat Kolankiewicz

**Affiliations:** ^1^ Nursing Program Regional University of the Northwest of the State of Rio Grande do Sul (UNIJUÍ) Ijuí Brazil; ^2^ Stricto Sensu Graduate Program in Comprehensive Health Care Regional University of the Northwest of the State of Rio Grande do Sul (UNIJUÍ) Ijuí Brazil; ^3^ Nursing Research, Innovation and Development Centre of Lisboa (CIDNUR), School of Nursing Universidade de Lisboa Lisbon Portugal; ^4^ Center for Innovative Care and Health Technology (ci Techcare) Polytechnic of Leiria Leiria Portugal; ^5^ Faculty of Medicine, Ciencias de la Salud, University of Santiago de Compostela Santiago de Compostela Spain; ^6^ Undergraduate Program in Medicine, Institutional Scholarship for Technological Development and Innovation (PIBITI) Regional University of the Northwest of the State of Rio Grande Do Sul (UNIJUÍ) Ijuí Brazil

**Keywords:** chronic illnesses, continuity of patient care, hospital discharge, transitional care

## Abstract

**Aim:**

To assess the relationship between sociodemographic and clinical characteristics and the quality of care transitions among individuals with chronic non‐communicable diseases after hospital discharge.

**Design:**

Cross‐sectional, single‐centre study.

**Method:**

Participants were people with chronic diseases who underwent clinical or surgical treatment and were discharged from a hospital in southern Brazil. Data were collected from March to July 2021. The study used clinical and sociodemographic characterisation instruments at the bedside; after hospital discharge, the care transitions measure‐15 (CTM‐15) was conducted by telephone. Descriptive and inferential statistical analyses were applied.

**Results:**

A total of 487 participants were included, with a mean age of 60.6 years; 67.4% were classified as having satisfactory care transitions. In the adjusted model, a higher likelihood of satisfactory transitions was observed among individuals who underwent surgical treatment (OR = 2.974; *p* < 0.001), those without family income (OR = 2.546; *p* < 0.001), White individuals (OR = 1.705; *p* = 0.027), and women (OR = 1.589; *p* = 0.042). Discharge with clinical devices was also associated with satisfactory transitions (OR = 1.245; *p* = 0.043). Conversely, individuals with family income between R$1100 and R$3299 showed a lower likelihood of satisfactory transitions, although this association was not statistically significant (OR = 0.724; *p* = 0.066).

**Conclusion:**

Clinical and sociodemographic characteristics significantly influence the quality of care transitions. The results highlight social and contextual factors and show that effective transitions require strategies that integrate clinical needs with the social determinants shaping the care experience. Transition practices that are sensitive to patients' social, cultural and economic contexts may help strengthen equity and safety in post‐discharge continuity of care.

**Implications for the Profession and/or Patient Care:**

These findings reinforce the need for individualised discharge planning, clear interprofessional communication and health education adapted to patients' literacy levels and social conditions. Such measures can improve safety, autonomy and treatment continuity for patients with chronic conditions and support professionals in developing context‐responsive care transition strategies.

**Reporting Method:**

This study followed the STROBE checklist for cross‐sectional studies.

**Patient or Public Contribution:**

Patients participated in the data collection by answering the care transitions measure‐15 after hospital discharge, directly contributing to the interpretation of the care transitions data.

## 1 Introduction

Non‐communicable chronic diseases (NCDs) are of non‐infectious origin and result from metabolic changes that compromise various levels of organisation in the human body, potentially leading to partial or total loss of function in organ systems. Their development occurs gradually and over a prolonged period, being influenced not only by individual lifestyle choices but also by environmental factors. This progressive nature creates windows of opportunity for preventive actions while also requiring continuous and systematic treatment over time (World Health Organization 2005).

In Brazil in the 20th century, people were affected by infectious diseases and part of the deaths occurred as a result (Buchalla et al. 2003). However, with the arrival of vaccines and changes in hygiene habits, Brazil's epidemiological scenario underwent changes and began to give rise to NCDs; they are considered the main cause of death and illness in the population and constitute an epidemic in Brazil (Buchalla et al. 2003).

Epidemiologically, they affect more than 700,000 people annually in the country; around 50% of the population was diagnosed with at least one NCD in 2019, and this is compounded by the alarming figure of 308,000 premature deaths (30 to 69 years of age) from such illnesses. In view of this scenario, we are currently experiencing a serious public health economic and social development situation in Brazil, as these diseases are responsible for more than half of all Brazilian deaths (Brasil 2023a).

This aligns with the Institute for Applied Economic Research states that, by the year 2030, the sustainable development goals (SDGs) aim to reduce premature mortality from non‐communicable diseases by one‐third through prevention and treatment, and to promote mental health and well‐being (Brasil 2023b).

As a result, it is necessary to find a way to facilitate the processes in health care networks (HCNs) through lines of care by defining actions and services that should be developed at the different points of care in networks (primary, secondary and tertiary levels). Therefore, the lines of care outline the users' therapeutic itinerary and must be clearly defined when the users enter at any point in the systems (Eleone et al. 2023). Strategic actions are important to foster the reorganisation of services and the strengthening of care for people with NCDs in primary health care (PHC) (Brasil 2021). Commonly, professionals at the primary and secondary levels do not have access to all the necessary information to provide continued care and do not understand the reasons why the users seek specialised attention.

This problem has led the World Health Organization to suggest intervention strategies and policies to consolidate person‐centred health services. In particular, the reorganisation of care models is recommended to adapt the response of health systems in order to combat possible fragmentation of care and disparate guidelines given by different professionals, which are perceived as ambiguous, confusing and having a high probability of errors and non‐adherence to therapeutic regimens (World Health Organization 2018).

Furthermore, it is important to consider the Care Transition Theory (Meleis 2007), which points out that in the transition process, diversities and complexities associated with changes in life, health, relationships and the environment that the person experiences in a unique and individual way must be considered. Personal conditioning aspects are associated with change, and their changes result from cultural beliefs and attitudes, socioeconomic situation, preparation and knowledge of the elderly and their families.

In this sense, the concept of transition requires nursing professionals to know the individual to be transitioned and their family members longitudinally, in order to facilitate and visualise situations that may generate instability. When promoting transitional care, the nurse values the patient by thinking about human maturation, seeking to achieve balance and stability (Meleis 2007), favouring person‐centred care.

In view of these issues, care transitions (CT) can be seen as a strategy to facilitate care processes. It is also defined as a set of strategies designed to ensure continuity of care in the referral and counter‐referral of patients between health services at different levels of care or at home (Coleman and Boult 2003; Eleone et al. 2023). In addition, CT aims to reduce the fragmentation of health care and ensure quality in the continuity of care.

It also seeks transdisciplinarity, helps to reduce unnecessary healthcare costs, contributes to the integration of health processes and reduces the number of adverse events and complications at more complex levels (Mendes et al. 2017; Morés 2021). CT encompasses the development of comprehensive care plans. It must rely on professionals with technical and scientific knowledge who can educate patients and caregivers during health and illness processes, besides talking and coordinating care management between PHC and specialised health care (SHC) (Coleman and Boult 2003).

A study carried out in Portugal found that transitional care contributed to improving quality of and access to care. It also observed: gains associated with persons and their families, particularly in terms of autonomy when returning home; reduced use of emergency services; a decrease in caregivers' anxiety; and coordination between different levels of care (hospital and primary care) (Pedrosa et al. 2022). In order to help create strategies for a safe transition and also to visualise where the weak points in this process lie, it is necessary to evaluate the performance of care lines in their care flows from the perspective of patients and their caregivers when they are discharged from secondary and tertiary care and go back to PHC. This assessment can be made with the care transitions measure‐15 (CTM‐15), developed by Coleman and Boult (2003) and validated for use in Brazil by Acosta et al. (2016).

A study in the Brazilian state of Rio Grande do Sul used the CTM‐15 instrument to assess CT in people living with chronic diseases. The results showed a satisfactory evaluation of most factors covered by the instrument, with averages above 70, such as: assured preferences, preparation for self‐management and understanding of medications. The care plan factor received the only unsatisfactory rating, with an average of 64.5. This shows that the SHC teams have not prioritised care plans in clinical practice, which can lead to discontinuity of care and increase the chances of returning to health services for avoidable reasons (Berghetti et al. 2023).

Furthermore, the CTM‐15 has proven to be an important tool for assessing the quality of CT plans, even though studies are still incipient. Most of the published studies have used the CTM‐15 to assess the quality of health services or to analyse the reliability and validation of the instrument. However, there are no studies in the literature identifying quality predictive factors for satisfactory CT plans, which is why this study was carried out. Denoting novelty in carrying out this study, given the robustness of its analysis in identifying predictive factors. This study aimed to assess the relationship between sociodemographic and clinical characteristics and the quality of care transitions among individuals with chronic non‐communicable diseases after hospital discharge.

## 2 Method

### 2.1 Study Design

This was a cross‐sectional study carried out in a 126‐bed general hospital of medium to high complexity, located in the northwest region of the State of Rio Grande do Sul, Brazil.

### 2.2 Participants and Recruitment

#### 2.2.1 Inclusion Criteria

Patients aged 18 years or older, hospitalised due to NCDs for a minimum period of 24 h, who were conscious and oriented at the time of bedside approach, and who could be contacted by phone after discharge were included in the study.

#### 2.2.2 Non‐Inclusion Criteria

Pregnant women, those with a prognosis of death, and individuals who were institutionalised in long‐term care facilities after discharge were excluded from the study.

### 2.3 Data Collection

Data collection was carried out between March and July 2021. During this period, 1112 patients were hospitalised. Of these, 151 did not meet the inclusion criteria and four declined to participate, resulting in 957 eligible individuals. Based on the total number of hospitalisations and assuming a 95% confidence level and an expected proportion of 50%, the minimum required sample size was estimated at 286 participants. However, the study included 487 individuals, resulting in an estimated maximum sampling error of 3.4%, which increased the precision of the findings.

Of the 957 eligible individuals, four declined to participate in the study. Additionally, 10 participants could not be contacted by telephone after discharge, 11 were transferred to other hospitals, and 14 had a prognosis of death and were therefore excluded according to the established criteria.

Data was collected at the bedside by a nurse Monday through Friday. Initially, patients received explanations of the purpose and procedures of the collection and were then asked to sign two identical copies of an informed consent form (ICF).

Data collection occurred in two stages: (1) in‐hospital assessment and (2) post‐discharge follow‐up.

#### 2.3.1 Sociodemographic Data

During hospitalisation, participants completed a structured interview that included sociodemographic variables such as sex, age, address, family income, education level, race/ethnicity and marital status.

#### 2.3.2 Clinical Data

Clinical variables were obtained through a review of medical records and included: reason for hospitalisation; hospitalisation unit; length of stay; number of hospital admissions in the previous 12 months; medications in continuous use; presence of clinical devices at discharge (e.g., catheters, drains, ostomy bags); previous and current diagnoses of chronic diseases.

#### 2.3.3 Post‐Discharge Data

Between 7 and 30 days after hospital discharge, the selected patients received phone calls to answer the CTM‐15 (Coleman et al. 2005), which was validated for use in Brazil by Acosta et al. (2016). It measures the quality of CT from hospital discharge to patients' place of residence or between different services from the perspective of patients and their caregivers (Coleman et al. 2005).

The 7 to 30‐day period is a methodological imperative from the instrument's author. This interval is critical because it allows the patient not only to experience the care transition but also to actively identify the challenges and successes of post‐discharge self‐management. This encompasses understanding the therapeutic plan, correct medication usage and the ongoing need for support at home (Coleman et al. 2005).

The instrument includes 15 questions and is divided into four sets of questions that measure the following factors: preparedness for self‐management (4, 5, 6, 8, 9, 10 and 11); understanding of medications (13, 14 and 15); assured preferences (1, 2 and 3); and care plan (7 and 12) (Coleman et al. 2005; Acosta et al. 2020). It is assessed using a five‐point scale: Does not know/does not remember/does not apply = 0; Strongly disagree = 1 point; Disagree = 2 points; Agree = 3 points; Strongly agree = 4 points. The formula applied to calculate the averages transforms the results obtained into scores from 0 to 100 (Coleman et al. 2005). The authors consider a score of 70 or more to be satisfactory (Acosta et al. 2020).

### 2.4 Data Analysis

Data analysis identified classification as satisfactory or unsatisfactory. The analysis was carried out with the Statistical Package for Social Sciences version 25.0 (SPSS Inc., Chicago, IL, USA, 2015), considering a significance level of 5%. Descriptive statistics were applied with absolute and relative distributions and measures of central tendency and variability, and the Kolmogorov–Smirnov test was used for the study of the symmetry of continuous distributions. When comparing continuous variables between CTM classifications, Student's *t*‐test for independent groups and Mann Whitney U (asymmetric distributions) were used. When the analyses involved categorical variables, the Pearson Chi‐square test (2) was used, complemented by the risk estimate using the odds ratio [95% CI].

The analysis involving the predictive/explanatory capacity for the CTM classification was investigated using the Binary Multiple Logistic Regression technique [Unsatisfactory (0) and Satisfactory (1)]. As independent variables, sociodemographic and clinical characteristics were listed that presented a minimum level of significance lower than or equal to 0.200 (*p* ≤ 0.200) in the bivariate analysis, when compared to the CTM classification. The selection of representative variables occurred using the conditional Backward method. To check the goodness of fit of the final logistic regression model, the Nagelkerke and Hosmer‐Lemeshow R2 estimators were considered. The probability of gradual entry of variables into the model was 0.05 and 0.10 for removal.

### 2.5 Ethical Considerations

All participants were informed about the study objectives during hospitalisation and, upon acceptance, signed the informed consent form. The study complied with Resolution 466/12 and was approved by the Research Ethics Committee under Opinion No. 4.479.127/2020.

## 3 Results

A total of 487 patients participated in the study. Regarding NCD status, 42.1% (*n* = 205) of the patients had one NCD; 30.6% (*n* = 149), two; 18.5% (*n* = 90), three; 8.6% (*n* = 42), four or more; and 81.3% (*n* = 396) of the individuals had been diagnosed with NCDs for more than a year. The average age of the entire sample was 60.6 years; 54.6% (*n* = 266) were over 60 years old; 53.8% (*n* = 292) lived in urban areas; 52.6% (*n* = 256) were female; 76.4% (*n* = 372) were white; and 60% (*n* = 291) were married or were in a stable relationship. As for education, 71.8 (*n* = 343) had attended an educational institution for up to 8 years.

Patients were evaluated according to the results of the CTM‐15 scale: unsatisfactory, 32.6% (*n* = 159); and satisfactory: 67.4% (*n* = 328). When the CTM classification was compared with regard to the variables that characterised the sociodemographic profile, a statistically significant difference was identified in relation to skin colour (*p* = 0.006), indicating that satisfactory classification on the CTM‐15 was associated with white patients, 80.4% (*n* = 262); while in cases where the classification was unsatisfactory, the association occurred with non‐white patients, 30.8% (*n* = 49). According to the odds ratio estimate, white patients were 1.82 times more likely to have a satisfactory rating when compared to non‐white patients.

Another result was observed for family income, which showed significant differences for the CTM‐15 classification (*p* = 0.008). It was found that patients with an unsatisfactory rating were significantly associated with the income range of R$1,100 to R$3,299,49.7% (*n* = 73); while in cases with a satisfactory rating, the association occurred with the absence of income, 12% (*n* = 41); and with the highest income range—R$3,300 or more, 53.3% (*n* = 170). Patients who stated they had no income were 1.53 times more likely to have satisfactory classification on the CTM‐15 when compared to the group with the highest income in the sample, regarding the risk estimate for a satisfactory classification. In addition, having a family income between R$1,100′0.00 and R$3,299.00 proved to be a protective factor for a satisfactory classification (OR: 0.54), that is, patients in this income range are more likely to have an unsatisfactory classification.

The other variables of the sociodemographic profile showed no significant differences when compared according to the CTM‐15 classification. As shown in Table 1.

**TABLE 1:** Sociodemographic characterisation of people living with NCDs, by CTM‐15 classification. RS, Brazil 2023.

Variables	Total da amostra	CTM Classification[*n* = 487]			
		Unsatisfactory (score < 70 points)	Satisfactory (score ≥ 70 points)	MD	*p*
	(*n* = 487)	(*n* = 159)	(*n* = 328)		
	*n* (%)	*n* (%)	*n* (%)	*n* (%)	
Sex					
Male	231 (47.4)	83 (52.2)	148 (45.1)	0	0.171^a^
Female	256 (52.6)	76 (47.8)	180 (54.9)		
Age (years)					
Average ± standard deviation	60.6 ± 17.2	6**1**.8 ± 17.7	60.3 ± 16.9	0	0.366^b^
Age group (years)					
From 18 to 39	59 (12.1)	18 (11.3)	41 (12.5)	0	0.930^a^
From 40 to 59	162 (33.3)	53 (33.3)	109 (33.2)		
60 years or more	266 (54.6)	88 (55.3)	178 (54.3)		
Skin colour					
White	372 (76.4)	110 (69.2)	262 (80.4)	2 (0.4%)	0.006^a^
Non‐white	113 (23.6)	49 (30.8)	64 (19.6)		
Schooling					
Until medium school	343 (71.8)	121 (76.1)	222 (69.6)	9 (1.8%)	0.232^a^
High school	111 (23.2)	33 (20.8)	78 (24.5)		
Higher education	24 (5.0)	5 (3.1)	19 (6.0)		
Marital status					
Married/common‐law marriage	291 (60.0)	96 (60.4)	195 (59.6)	1 (0.2%)	0.875^a^
Not married	195 (40.0)	63 (39.6)	132 (40.4)		
Family income					
No income ou Without income	53 (11.4)	12 (8.2)	41 (12.9)	21 (4.3%)	0.008^a^
From R$ 1.100 to R$ 3.299	181 (38.8)	73 (49.7)	108 (33.9)		
R$ 3.300 or more	232 (49.8)	62 (42.2)	170 (53.3)		

Abbreviation: MD, missing data.

^a^ Pearson's chi‐square test of independence.

^b^ Student's *t*‐test for independent groups.

Clinical information in line with the CTM‐15 classification identified a statistically significant association with treatment (*p* < 0.001), where patients with surgical treatment were associated with a satisfactory classification, 36.3% (*n* = 119). With regard to risk estimation, patients undergoing surgical treatment were 2.48 times more likely to have a satisfactory classification than those undergoing clinical treatment.

Length of hospitalisation also proved to be sensitive to the CTM classification (*p* = 0.033), where the average time for patients with a satisfactory classification [4 (3–8)] was significantly shorter than that of cases with an unsatisfactory classification [6 (3–10)]. This result indicates that patients with shorter hospital stays have a higher chance of a satisfactory classification (OR = 0.97; 95% CI: 0.95–0.99).

Discharge with the presence of clinical artefacts was significantly associated with a satisfactory rating, 49.7% (*n* = 163) [*p* = 0.003], indicating that patients who used clinical artefacts were 1.21 times more likely to have a satisfactory rating than those who did not use artefacts.

The other clinical characteristics related to treatment, such as chronic diseases and hospitalisations, Transitional care interventions and readmissions did not show statistically significant results according to CTM classification, as shown in the Table 2.

**TABLE 2:** Characterisation from clinical information of the sample, by the CTM‐15 classification, RS, Brazil. 2023.

Variables	Total da amostra	CTM Classification [*n* = 487]			
		Unsatisfactory (score < 70 points)	Satisfactory (score ≥ 70 points)	MD	*p*
	(*n* = 487)	(*n* = 159)	(*n* = 328)		
	*n* (%)	*n* (%)	*n* (%)	*n* (%)	
Treatment					
Surgical	149 (30.6)	30 (18.9)	119 (36.3)	0	< 0.001^a^
Clinical	338 (69.4)	129 (81.1)	209 (63.7)		
Number of chronic diseases					
1Uma	205 (42.2)	63 (39.6)	142 (43.4)	1 (0.2%)	0.647^a^
2Duas	149 (30.7)	49 (30.8)	100 (30.6)		
3Três	132 (27.2)	47 (29.6)	85 (26.0)		
Length of chronic diseases					
Up to 12 months	35 (8.1)	7 (4.9)	28 (9.8)	56 (11.5%)	0.096^a^
From 1 to 6.5 years	168 (39.0)	64 (44.4)	104 (36.2)		
More than 6.5 years	228 (52.9)	73 (50.7)	155 (54.0)		
Length of chronic diseases					
Average ± standard deviation	4.3 ± 1.6	4.39 ± 1.5	4.2 ± 1.7	40 (8.2%)	0.902^b^
Median (1st–3rd quartile)	5.0 (3.0 – 6.0)	5 (3–5)	5 (3–5)		
Length of hospitalisation					
Average ± standard deviation	7.3 ± 8.2	8.5 ± 9.2 (1–65)	6.7 ± 7.7 (1–83)	0	0.033^b^
Median (1st–3rd quartile)	5 (3–8)	6 (3–10)	4 (3–8)		
Length of hospitalisation					
From 1 to 5 days	269 (55.2)	79 (49.7)	190 (57.9)	0	0.141^a^
From 6 to 14 days	166 (34.1)	58 (36.5)	108 (32.9)		
Above 14 days	52 (10.7)	22 (13.8)	30 (9.1)		
Post‐discharge clinical artefacts					
Yes	219 (45.0)	56 (35.2)	163 (49.7)	0	0.003^a^
No	268 (55.0)	103 (64.8)	165 (50.3)		
Received intervention from TC					
Yes	139 (28.8)	45 (28.3)	94 (29.1)	5 (1.0%)	0.855^a^
No	343 (71.2)	114 (71.7)	229 (70.9)		
Readmitted					
Yes	33 (6.8)	15 (9.4)	18 (5.5)	0	0.104^a^
No	454 (93.2)	144 (90.6)	310 (94.5)		

Abbreviation: MD, missing data.

^a^ Pearson's chi‐square test.

^b^ Mann–Whitney U test.

According to the results obtained, Table 3 shows the initial (saturated) and final (reduced) models; the latter was established in four steps/stages using the backward conditional selection method. Analysis using the Cox & Snell and Nagelkerke tests indicated values of 0.184 and 0.222, respectively.

**TABLE 3:** Binary logistic regression model to estimate the effects on the CTM‐15 classification (satisfactory) by clinical and sociodemographic characteristics, RS, Brazil 2023.

Independent variables	Modelo inicial			Modelo final			
	aOR	IC 95% OR	*p*	aOR	IC 95% OR		*p*
Length of hospitalisation							
Above 14 days	1000		0.354				
From 1 to 5 days	1.473	0.731–2.967					
From 6 to 14 days	1.111	0.541–2.283					
Post‐discharge clinical artefacts [ yes]	1.245	1.019–2.007	0.043				
Sex, Female	1.574	1.029–2.407	0.036	1.589	1.126–2.713		0.042
Skin colour, White	1.777	1.076–2.937	0.025	1.705	1.157–3.931		0.027
Education							
Medium school	1000		0.510				
Higher education	1.051	0.580–1.902					
High school	2.248	0.571–8.852					
Family income							
R$ 3.300 or more	1000		0.066	1.0			< 0.001
Does not have	2.971	1.188–4.805		2.546	1.740–4.119		
From R$ 1.100 to R$ 3.299	0.920	0.467–1.812		0.724	0.523–0.966		
Surgical treatment	1.795	0.940–3.428	0.83	2.974	1.644–4.588		< 0.001
Age (years)	0.994	0.980–1.009	0.163	0.865	0.755–0.986		0.019

*Note:* Initial Model—Nalgelkerke R2 0.222; Hosmer‐Lemeshow proof (Chi‐Square (8) = 4.220; *p* = 0.837); Cox & Snell: 0.184; Overall hit ratio—confusion matrix: 68.7%. Final model—Nalgelkerke's R2 0.217; Hosmer‐Lemeshow test (Chi‐Square (8) = 10.806; *p* = 0.213); Cox & Snell: 0.197; Overall hit ratio—confusion matrix: 70.3%.

*Source:* Elaborated by the author.

Cox & Snell indicated that in the final model, 18.4% of the variations in the log of the odds ratio are explained by the set of independent variables. With a significance similar to that of the coefficient of determination, Nagelkerke's estimator considered that the model was able to explain 22.2% of variations in the CTM classification.

In the model summary, it was observed that there were no significant differences between the model estimates and the actual sample classifications for unsatisfactory and satisfactory; that is, the model has a significant predictive capacity. There is evidence that, in the confusion matrix, the total hits of 70.3% were representative in the study, since the model correctly classified 58.9% of cases with unsatisfactory classification and 81.7% of patients with satisfactory classification.

By analysing the effects estimated by the model in Table 3, it was found that in the initial model some variables lost their explanatory power, as well as strength of association, compared to the adjustment of the other explanatory variables belonging to the model. Those that are no longer representative to predict the CTM classification are: length of hospitalisation, post‐discharge clinical artefacts and education.

Therefore, surgical treatment stood out as the most representative variable to predict/explain satisfactory CTM classification [OR: 2.974; 95% CI: 1.644–4.588]; followed by the absence of family income [OR: 2.546; 95% CI: 1.740–4.119]; being white [OR: 1.705; 95% CI: 1.157–3.931]; being female [OR: 1.589; 95% CI: 1.16–2.7713]; and using post‐discharge clinical artefacts [OR: 1.245; 95% CI: 1.019–2.007] (Table 3).

## 4 Discussion

The results of this study showed that the clinical and sociodemographic factors that influenced CT also stood out as the most representative variables in explaining the satisfactory CTM‐15 classification. They were: surgical treatment, white skin colour, female sex, lack of per capita income and discharge with clinical artefacts.

With regard to treatment modality, patients who underwent surgical treatment were better prepared for self‐management at home, and this may be associated with satisfaction with CT (Costa et al. 2020). With the aim of improving the CT of elderly patients at home, a study carried out by Costa et al. (2020) used the creation of educational material as a strategy to facilitate patient self‐management, in order to overcome the lack of knowledge about the post‐surgical process. The educational material was given to patients and caregivers by nurses. They contained important information, such as general care, management of surgical wounds and information about return visits to outpatient clinics (Costa et al. 2020).

This type of strategy supports not only patients' self‐management, but also the construction of discharge plans. In this study, the implementation of discharge plans with caregivers showed a 25% reduction in readmissions in 90 days, and 24% in 180 days (Costa et al. 2020). The use of high‐quality plans promotes knowledge of the patient as well as family members and/or caregivers regarding the organisation of care. By having an understanding of how to manage devices, medications and other necessary instruments, they are able to perform care more safely and effectively (Costa et al. 2020).

In line with the results of this research, a study carried out in the surgical clinic of a teaching hospital in João Pessoa (Brazil) found a gap in the relationship between nurses and patients that prevented information from being passed on to patients during the discharge process. According to the study, the professionals did not provide the necessary information with accessibility and did not let caregivers participate in the conversations. It ended up preventing the creation of bonds, a situation in which patients could not ask questions and express their concerns, making it difficult to understand which type of care patients and caregivers should provide at home in order to achieve a full recovery (Martins et al. 2015).

The complexity of health‐disease processes and the transitions experienced by helpless people is a challenge for health systems. In many cases, elderly people discharged from hospitals become even more care‐dependent. Due to their clinical situation, they cannot return to prehospitalisation levels of function, proving that there is a need to prepare caregivers and ensure self‐care when they return home (Thawanna et al. 2021).

These aspects lead us to reflect on our professional attitudes, and some sort of change in attitude in the clinical sphere is essential. It is also suggested that nurses should plan and document what is offered as guidance at the time of hospital discharge for a better understanding of patients and caregivers and for the PHC service, which will receive the counter‐referral process (Martins et al. 2015).

Also during hospitalisation, it is necessary to promote caregivers' adaptation to their new roles (transition to informal caregiver) in order to acquire the knowledge and skills needed to ensure continuity of care and the ability to manage self‐care (Thawanna et al. 2021). These interventions should be validated or continued upon return home, as they impact the management of the patient's therapeutic regimen and the well‐being of the caregiver.

Regarding skin colour, studies have shown that structural and institutional racism still affects access to health services and the quality of care provided, which leads to unequal results between races (Smith et al. 2021). The satisfaction of white people can be attributed to those issues.

One study examined the relationship between race, ethnicity and hospital discharge among people hospitalised for diabetes‐related conditions, showing that Black patients were less frequently counter‐referred to PHC compared to non‐Black patients; moreover, when counter‐referral did occur, Black patients were also less likely to receive home care from PHC teams in situations involving complications (Smith et al. 2021). The same study also found that Black patients received superficial instructions on how to perform self‐care at home after discharge and presented a greater need for readmissions within 2 weeks. These issues reflect inadequate discharge planning and weak shared coordination of care, which, if implemented in a timely and equitable manner, could have reduced the risk of adverse outcomes (Smith et al. 2021).

As for sex, a finding of satisfaction may be due to the fact that, socially, women tend to take better care of their health than men and this difference between the sexes affects the way men and women use the National Health System (SUS) (Costa‐Júnior et al. 2016). Future studies should explore this association, as well as the sex issues associated with informal caregivers and whether they interfere with transitional care.

A study by Costa‐Júnior et al. (2016) tried to understand the perceptions of health professionals about the implications of sex in health care in the context of outpatient and inpatient care. Health professionals have reported differences in care and attitudes of male and female patients. Such differences are attributed to biological and social factors. Women tend to seek health services more often, while men avoid prevention and give up treatment when in a serious situation. When hospitalised, men tend not to accept care, which leads to longer hospital stays than women. The consequences are worse health conditions and greater chances of unfavourable outcomes (Costa‐Júnior et al. 2016).

In addition, health professionals' ideas about sex can influence the way they provide care. In the study, nurses showed a broader understanding of sex, seeing it as a historical construction of a political and social nature, while doctors tended to justify sex differences based on organic and anatomical factors. This can lead to inequalities in care and the quality of the services offered, impacting the professional‐patient relationship. It is therefore important to consider sex issues in the training of health professionals and in the provision of health services, to ensure equity and comprehensiveness at different levels of health care (Costa‐Júnior et al. 2016).

When it comes to family income, the results of the present study show that not having an income is a factor in satisfactory classification. This result contradicts what we have found in the literature regarding the social inequality experienced by people with low or no family income. Normally, low‐income individuals have worse health outcomes compared to people with higher family incomes, who have better access to health services and can obtain diagnoses of CNCDs more quickly and accurately, which directly influences the control of the treatment (Antunes et al. 2018). Higher family income is often also combined with a higher level of education (Antunes et al. 2018), which directly affects how to understand these patients during treatment.

This contrasting result may be associated with the fact that, in the present study, the CTM‐15 questionnaire was administered to some people with no income. When responding to the questionnaire, they wished to express gratitude for the care received and sometimes ended up agreeing with all the statements in order to please the interviewers—an example of gratitude bias, which occurs when participants evaluate services or professionals excessively positively because they feel grateful or indebted, thereby distorting the accuracy of their responses. This may have influenced the result.

With regard to individuals who were discharged with medical devices, satisfaction may be related to better discharge preparation and, consequently, improved self‐management of their care. When patients receive device‐specific instructions, they tend to adhere more effectively to home care, which promotes greater autonomy and strengthens self‐care (Oliveira et al. 2022).

It is essential that health professionals employ health education strategies during discharge preparation, not only for patients but also for caregivers. The aim is to guarantee rehabilitation possibilities and avoid adverse events, and the possibility of readmission due to avoidable issues (Bandeira et al. 2020).

As mentioned above, CT is only effective for certain sociodemographic and clinical characteristics, so we need to organise interventions so that CT can be accessed by a multitude of different patient profiles. Initially, it is necessary to invest in the training of health professionals, even during their education/training. It is essential to include content that encompasses diversity and intersectionality, so that these professionals can understand and respect the differences between and needs of each patient, regardless of their race, sex or family income, among others (Machin et al. 2022).

Secondly, we can use some strategies identified in the literature to create safe and effective CT in healthcare environments. The availability of telephone contact between SHC and PHC nurses is proving to be effective in strengthening communication between services while enabling continuity of treatment at different network levels (Day et al. 2016). Upon hospital discharge, it is essential to establish a scheduling flow for return appointments to ensure follow‐up on these patients (Acosta et al. 2020). Furthermore, educational actions that consider health literacy levels are essential, so that users and family members understand the care for their decision‐making in post‐discharge care (Gheno et al. 2024).

Setting up active post‐discharge surveillance programmes may help monitor patients after hospital discharge and identify complications or health problems at an early stage. This monitoring can be carried out through telephone contact or telemedicine, aiming to ensure proper recovery and reduce the likelihood of further hospitalisation (Trindade et al. 2022).

Using a standardised enhanced recovery protocol in the form of a set of guidelines and clinical practices that optimise patient recovery after surgery may be capable of significantly reducing hospital readmissions and improving patient experience (Trindade et al. 2022).

In order to address these issues, it is necessary to have institutional organisations that promote CT within PHC and its healthcare networks, with health professionals who are established, committed and knowledgeable about the processes that involve safe and effective CT (Danielle et al. 2025; Rodrigues et al. 2022). In other studies, we observe the continuous insertion of nursing professionals, also known as liaison nurses or navigators, in transition processes. Their role is to coordinate post‐discharge care while guiding patients within healthcare networks, facilitating access to services and identifying needs (Rohsig et al. 2019).

Regarding the limitations of the study, we can mention that it was carried out in a single hospital, characterising it as a single‐centre study and the possibility of selection bias, as patients were recruited for convenience. These characteristics can lead to non‐generalisable results. Therefore, it is suggested that more studies be carried out in different health contexts and geographic regions, using probabilistic sampling techniques or expanding recruitment to include more diverse and representative populations, minimising the possibility of selection bias. This study also contributes to the production and dissemination of knowledge about CT, which is still in its infancy among literary productions and needs to be improved nationally.

## 5 Conclusion

The findings of this study show that sociodemographic and clinical characteristics significantly influence the quality of care transitions among individuals living with chronic conditions. The higher likelihood of satisfactory evaluations among patients who underwent surgical treatment, women, White individuals, those without family income and those discharged with medical devices highlights structural inequalities that shape the post‐discharge experience. These results indicate that care transitions are deeply conditioned by social determinants that affect patients' understanding of discharge instructions, capacity for self‐management and continuity of treatment.

It is essential to adopt socially sensitive transition strategies, incorporating clear communication, patient‐centred discharge planning and health education actions that account for literacy levels and sociocultural diversity. Strengthening coordination between hospital and primary care services and ensuring equitable access to information are key steps to improving care quality. Multicenter studies with more representative samples are needed to deepen understanding of these determinants and to guide more equitable and responsive care transition models.

## Author Contributions

**Mardhjorie dos Santos Seidler:** conceptualisation, data curation, investigation, methodology, project administration, resources, supervision, validation, visualisation, writing – original draft, writing – review and editing. **Larissa Berghetti:** conceptualisation, data curation, investigation, methodology, project administration, resources, supervision, validation, visualisation, writing – original draft, writing – review and editing. **Letícia Y. Castro:** writing – review and editing. **Francini de Oliveira Rodrigues:** writing – review and editing. **Cristina Lavareda Baixinho:** writing – review and editing. **Manuel Portela Romero:** writing – review and editing. **Amanda Caroline Mélo da Rosa:** writing – review and editing. **Adriane Cristina Bernat Kolankiewicz:** conceptualisation, data curation, investigation, methodology, project administration, resources, supervision, validation, visualisation, writing – original draft, writing – review and editing. **Letícia Flores Trindade:** conceptualisation, data curation, investigation, methodology, project administration, resources, supervision, validation, visulisation, writing – original draft, writing – review and editing.

## Funding

This work was supported by Conselho Nacional de Desenvolvimento Científico e Tecnológico, 301694/2025‐7.

## Ethics Statement

Research in line with Resolution 466/12, approved by the Research Ethics Committee under Consolidated Opinion 4.479.127/2020.

## Conflicts of Interest

The authors declare no conflicts of interest.

## Data Availability

The data that support the findings of this study are available from the corresponding author upon reasonable request.
